# The influence of natural polymers on loratadine's solubility and dissolution profiles

**DOI:** 10.25122/jml-2023-0529

**Published:** 2024-03

**Authors:** Hussein Alkufi, Suad Lateef Ibrahim, Lina Salim Hussein

**Affiliations:** 1 Department of Pharmacognosy, College of Pharmacy, University of Thi-Qar, Nasiriyah, Iraq; 2 Department of Clinical Laboratory Science, College of Pharmacy, University of Kufa, Kufa, Iraq

**Keywords:** loratadine, natural solid dispersions, sodium alginate, hyaluronic acid, xyloglucan

## Abstract

Second-generation tricyclic H1 antihistamine loratadine (LTD) has a high permeability, low water solubility, and an oral absorption rate dependent on the rate at which it dissolves in the gastrointestinal tract. One approach suggested for improving the drug's solubility and rate of dissolution is natural solid dispersion (NSD). The present study evaluated the use of hydrophilic natural polymers, sodium alginate (SA), hyaluronic acid (HA), and xyloglucan (XG), in natural solid dispersion to enhance LTD solubility and dissolution rate. A total of 12 formulations comprising varied drug-to-polymer ratios were produced and analyzed for percentage yield, water solubility, and in vitro dissolution rate. The solubility of LTD was improved in all formulations. Excellent results were achieved with NSD1 (LTD: SA 1:0.25), with a high yield (99%), superior solubility (0.187) compared to pure loratadine (0.0021), and a speedy dissolution rate (98%) within 30 minutes. These studies suggest natural polymers like SA, HA, and XG can considerably increase LTD solubility. When introduced into NSD, these polymers effectively augment LTD dissolving rates, presenting attractive prospects for better bioavailability and therapeutic efficacy.

## INTRODUCTION

Drug solubility measures a drug's ability to dissolve in a certain liquid at a particular temperature. To achieve the intended pharmacological impact in the bloodstream, determining the ideal medicine concentration is essential. Developing novel drugs for pharmaceutical use poses a significant challenge, namely ensuring that medications exhibit solubility at the absorption site [1–3]. Natural solid dispersion (NSD) is used to increase the bioavailability of substances that are poorly soluble in aqueous media. In this method, the medication is combined with an inert substance to increase its solubility and effectiveness. The positive qualities of natural polymers, such as ample supply, biodegradability, and sustainability, are gaining significant interest. In contrast to synthetic polymers, their versatility and reduced occurrence of identified negative effects have piqued interest in a variety of culinary and medical applications. Many natural polymers have been used in nanosuspension to enhance drug solubility and dissolution profile, depending on their high water solubility. Hyaluronic acid (HA), xyloglucan (XG), and sodium alginate (SA), as examples of natural polymers, have been used in wound healing according to their therapeutic activity [4–6]. HA is a natural linear polysaccharide with a molecular weight of 900,000 to 1,000,000 Da and different medical uses. These uses include tissue engineering, medicine administration, ophthalmology and plastic surgery, and osteoarthritis treatment [7–9]. Sodium alginate is synthesized from sodium alginic acid, a polyanionic polysaccharide, and has a molecular weight of 10,000–15,000 Da. Depending on the surrounding pH, SA forms a gel layer that acts as a barrier to protect the gastrointestinal tract (GIT). Water penetration into the dosage form enhances surface hydration and gel formation when it enters the stomach, possibly assisting in the preservation of the stomach lining [10,11]. Xyloglucan from tamarind seeds is a naturally occurring neutral hemicellulose with a molecular weight of 52,350 Da that is well-known for its ability to form barriers and protective coatings [12,13]. In an effort to develop pharmaceutical formulations, solvent evaporation processes are used to enhance aqueous solubility. This process entails dissolving an inadequately water-soluble pharmaceutical medication in a suitable solvent and then allowing the solvent to evaporate to generate more soluble drug particles. The method starts by identifying a suitable solvent that can dissolve the medication and be utilized with the right dosage calculation. Subsequently, the drug is dissolved in the solvent, resulting in a solution or suspension. To increase the solubility enhancement technique, a range of criteria are considered, including solvent viscosity, polarity, and volatility. Once the drug has entirely dissolved or diffused into the solvent, it is evaporated under precisely regulated circumstances, such as pressure, temperature, and airflow. The drug particles nucleate and precipitate when the solvent evaporates, generating micro/nanocrystals—fine particles with better solubility and a greater surface area. The solvent evaporation strategy offers various advantages for increasing drug solubility, including its suitability for industrial-scale manufacturing, fundamental principles, and scalability. Furthermore, stable formulations and consistent dosing can be assisted by the production of homogenous particle sizes through controlled drug particle precipitation. In addition, the solvent evaporation technique can be utilized with other techniques, such as surface modification or polymer encapsulation, to change drug release kinetics and increase drug solubility [14–16]. Loratadine is a second-generation tricyclic H1 antihistamine that works by reducing the body's reactivity to histamine or allergens in the nasal and ocular areas, thereby relieving symptoms of seasonal allergies, itching, and watery eyes. LTD is classified as a class II medication under the Biopharmaceutical Classification System (BCS) and commonly faces issues related to poor solubility and dissolution, potentially inhibiting its uptake [17]. This decrease in bioavailability could make therapy less effective. It is considered that natural polymers like XG, HA, and SA have gastroprotective effects. This study aimed to develop naturally occurring solid dispersions of LTD and investigate their impact on the solubility and dissolution rate of the material.

## MATERIAL AND METHODS

For this study, LTD and HA (both >99% purity) were purchased from Hyperchem, xyloglucan (purity >95%) from Haihang Industry, sodium alginate (purity >99%) from Himedia Laboratories, and 99.9% pure methanol from Merck.

### Preparation of natural solid dispersion of loratadine

The solvent evaporation method was employed to create natural solid dispersions of LTD, as detailed in Table 1. The natural polymers SA, XG, and HA were utilized in this process. The following steps were executed:

**Table 1:** Composition of several NSD formulae of LTD

Formula Code	LTD (g)	SA (g)	XG (g)	HA (g)
NSD1	0.5	0.25		
NSD2	0.5	0.5		
NSD3	0.5	1		
NSD4	0.5	1.5		
NSD5	0.5		0.25	
NSD6	0.5		0.5	
NSD7	0.5		1	
NSD8	0.5		1.5	
NSD9	0.5			0.25
NSD10	0.5			0.5
NSD11	0.5			1
NSD12	0.5			1.5

Each polymer was separately dissolved in 5 milliliters of methanol using a stirrer with a magnet until a clear solution was produced. LTD was added to each polymer solution, and stirring continued for 5 minutes. Precise drug-to-polymer ratios (by weight) were computed based on the viscosity of the polymeric solution. Subsequently, the solvent was permitted to evaporate at room temperature. The resulting dried mass was crushed and passed through a No. 20 screen to ensure a homogeneous particle size distribution. Finally, the treated materials were stored in a desiccator for further investigation [18,19].

### Evaluation of NSD

#### Assessment of solid dispersion percentage yield (PY %)

The following formula was used to determine the formulated LTD solid dispersions percentage yield (PY %):


$$\mathrm{Pr}actical\;Yield\;(\%)=\frac{Mass\;of\;SD\;re\mathrm{cov}ered\;(\mathrm{Pr}actical\;mass)}{Mass\;of\;polymer\;and\;drug\;used\mathrm{in}\;formulation\;(Theoretical\;mass)}\times 100$$

This equation allowed the computation of the % yield for each given solid dispersion type by comparing the real weight of the achieved SD to the theoretically calculated weight [20].

### Determination of saturation solubility

Saturation solubility was determined through the following procedures:

10 milliliters of deionized water were acquired, and separate additions of LTD and NSD were introduced. The samples were subsequently placed in a water bath shaker at 25°C for 48 hours. After incubation, the samples were taken from the water bath shaker. Subsequently, the solutions were diluted appropriately and filtered using a 0.45 µm filter syringe. The concentration and solubility of LTD were measured using a UV spectrophotometer calibrated to 244 nm. The entire procedure was repeated three times to ensure the accuracy of the results [20,21].

### In-vitro dissolution studies of pure LTD and NSD

Following the outlined protocols, in-vitro dissolution investigations were conducted for pure LTD and NSD. The dissolution medium was prepared with 0.1 N HCl in 900 milliliters. After accurately weighing 10 mg of LTD and the equivalent of 10 mg of LTD in NSD, the materials were introduced into a USP type 2 dissolution apparatus. The dissolution test was conducted at 37 ± 0.5°C for 1 hour, stirring at 75 rpm. 5 milliliters of the dissolving medium were withdrawn at regular intervals, replaced with fresh dissolving medium, and then filtered. The dissolved content of LTD was measured at 280 nm using spectrophotometry. Each dissolution test was performed thrice, and the similarity factor (f2) was employed to compare the dissolution profiles [22,23].

## RESULTS

### The percentage yield of the prepared NSD

To assess the efficiency of the formulation method, it is essential to determine the percentage yield of the prepared formulation. Table 2 demonstrates all the NSD formulations.

**Table 2:** Percentage yield of NSD

Formula code	Percentage yield (%) ± SD*	Formula code	Percentage yield (%) ± SD*
NSD1	99 ± 0.01	NSD7	98 ± 0.02
NSD2	98.5 ± 0.22	NSD8	96 ± 0.02
NSD3	99 ± 0.12	NSD9	95 ± 0.01
NSD4	99.5 ± 0.34	NSD10	96 ± 0.03
NSD5	97 ± 0.02	NSD11	96 ± 0.01
NSD6	99 ± 0.33	NSD12	95.5 ± 0.06

* mean ± standard deviation (SD), n=3

### Saturation solubility of LTD NSDs

The findings of saturation solubility experiments of LTD NSDs are shown in Table 3.

**Table 3:** Comparative analysis of saturation solubility for LTD and NSDs in various natural polymers at 25°C

Formula code	Saturation solubility mg/ml Mean ± SD*	Formula code	Saturation solubility mg/ml Mean ± SD*
LTD	0.0021 ±0.001	NSD7	0.0143 ± 0.053
NSD1	0.187 ± 0.0211		
NSD2	0.2052 ± 0.123	NSD8	0.0131 ± 0.055
NSD3	0.1759 ± 0.002	NSD9	0.0586 ± 0.0211
NSD4	0.1642 ± 0.023	NSD10	0.0967 ± 0.098
NSD5	0.0146 ± 0.005	NSD11	0.0615 ± 0.0651
NSD6	0.0175 ± 0.023	NSD12	0.0322 ± 0.04322

Note: All measurements were taken in distilled water with the polymer ratio at 25°C

* mean ± standard deviation (SD), n=3

### In-vitro dissolution studies

**Figure 1:**
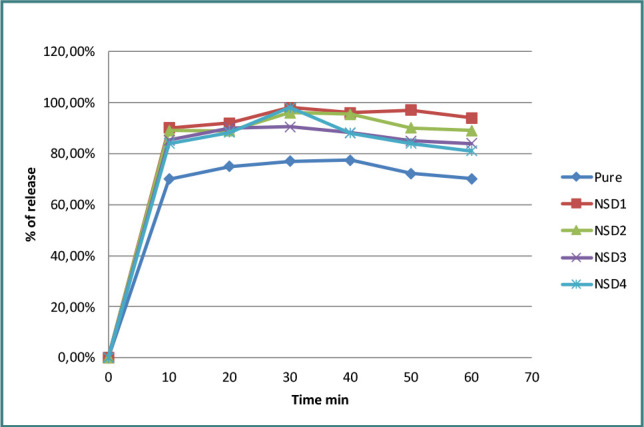
Release profiles of pure LTD and NSD formulations in 0.1 N HCl at 37 ± 0.5°C

For our in-vitro dissolution studies, NSD1, NSD2, NSD3, and NSD4 were selected for further examination due to their significant enhancement of LTD solubility. SA was used as a polymer in these formulations, with varying drug-to-polymer ratios (1:0.25, 1:0.5, 1:1, and 1:1.5). As seen in Figure 1, all these formulations NSD1, NSD2, NSD3, and NSD4 showed better dissolution rates in 0.1 N HCl compared to the pure medication. The similarity factors (f2) recorded for these formulations were 30.5, 28.44, 34.1, and 39.9, respectively.

## Discussion

Determining the percentage yield of the prepared formulation is crucial for assessing the efficiency of the formulation method. As shown in Table 2, all NSD formulations had high yields, ranging from 95% to 100%. Any minor losses observed were primarily attributed to the sieving or preparation steps. These results suggest that the solvent evaporation method, in conjunction with the selected components, is well-suited for these preparations. The ability of SA, HA, and XG to enhance the solubility of LTD was found to differ significantly (*P* <0.05). The order of efficacy in enhancing LTD solubility among different polymers can be ranked as follows: SA > XG > HA. This sequence was attributed to the special qualities of these polymers. For instance, the strong hydrophilicity of SA leads to rapid hydration of particles in the solid dispersion mixture upon contact with water. Despite no discernible difference between the four varied ratios (*P* >0.05), the wetting effect of SA, even in small quantities, may be responsible for the enhanced solubility of LTD in the solid dispersion. Higher polymer concentrations increase viscosity, preventing further solubility gains, explaining this result [14]. The enhanced solubility of LTD when combined with HA is attributed to the large, water-soluble molecules, which are capable of forming a highly viscous solution even at low concentrations. Viscosity increases with higher polymer concentrations, likely due to heightened hydrogen bonding potential and increased polymer chain entanglement. However, this may lead to a decrease in the solubility of LTD [24,25]. The solubility-increasing effect of XG, a water-soluble polymer, suggests its suitability for enhancing solubility in NSD synthesis. When comparing the drug-to-polymer ratios of 1:1 and 1:0.25, increasing the amount of XG did not result in a higher solubility. This may be related to the creation of a viscous barrier in the aqueous solution, preventing the hydration of LTD particles [13,26,27]. Remarkably, at the 30-minute point, NSD1 displayed the highest proportion of released LTD (98%), exceeding NSD2 (96%), NSD3 (90%), NSD4 (89%), and pure LTD (77%). The improved drug solubility in solid dispersions (SDs) is related to the smaller particle size of the drug in NSDs. Furthermore, it is hypothesized that the enhanced wettability and dispersibility of the medication in a hydrophilic polymer contribute to its dissolving profile [28]. It is worth noting that NSD1 and NSD2 exhibited a similar dissolution profile, with a similarity factor (f2) of 70.5. Earlier findings suggested that a decrease in the rate of LTD release was associated with an increase in SA. This outcome can be elucidated by the formation of a thicker viscous layer, hindering the diffusion of the drug from the NSD and acting as a barrier in the dissolving media. Consequently, there was a delay in the breakdown of the drug [29].

## CONCLUSION

The manufacture of LTD as a solid dispersion employing the solvent evaporation method, together with the utilization of SA, a naturally hydrophilic polymer, at a ratio of 1:0.25 (drug to polymer), resulted in enhancements to the solubility and dissolution rate of LTD. These advantages can be attributed to the reduced crystallinity of the medication and its improved wettability.

## Personal thanks

The authors thank the College of Pharmacy -University of Thi-Qar for providing the required facilities for this study.
